# Marijuana Use and Adherence to Smoking Cessation Treatment Among Callers to Tobacco Quitlines

**DOI:** 10.5888/pcd17.200110

**Published:** 2020-09-10

**Authors:** Kelly M. Carpenter, Alula J. Torres, Erica E. Salmon, Beatriz H. Carlini, Katrina A. Vickerman, Gillian L. Schauer, Terry Bush

**Affiliations:** 1 Center for Wellbeing Research, Optum, Minneapolis, Minnesota; 2 Alcohol and Drug Abuse Institute, University of Washington, Seattle, Washington; 3 University of Washington, School of Public Health, Seattle, Washington

## Abstract

**Introduction:**

Tobacco kills over half a million adults annually in the United States. Most smokers want to quit, and over 400,000 call state-funded quitlines for help each year. Marijuana use among tobacco users is common and may impede quitting, but co-use rates among quitline callers are unknown. The purpose of our observational study was to describe marijuana use among quitline callers in states with legalized marijuana.

**Methods:**

Participants were 1,059 smokers aged 21 or older from Oregon, Alaska, and Washington, DC, who called quitlines from September through December 2016. Data on quitline callers’ demographics, tobacco and marijuana use, and quitline use were collected. We used χ^2^ and regression analyses to compare marijuana users with nonusers on demographic characteristics and quitline use.

**Result:**

Among quitline callers in our study, 24% reported using marijuana in the past 30 days: 28.9% in Alaska, 16.7% in Washington, DC, and 25.0% in Oregon (*P* = .009). Current users, compared with non-users (n = 772), were less likely to be women (48.4% vs 62.0%, respectively, *P* < .001). Current marijuana users were less likely to be given nicotine replacement therapy (68.4%) than current nonusers (74.1%) (*P* < .001), but more likely to complete 3 or more counseling calls (*P* = .005). Of those who used marijuana in the past 30 days, 62.3% used marijuana on 1 to 19 days, 9.0% used on 20 to 29 days, and 28.7% on all 30 days. Among current marijuana users, the percentage who wanted to quit or reduce marijuana use (42.6%) was higher in Alaska (54.6%) and the District of Columbia (56.8%) than in Oregon (37.9%), *P* = .03.

**Conclusion:**

One in 4 quitline callers reported past 30-day marijuana use. Given that nearly half (43%) wanted to reduce marijuana use, addressing co-use may be an important addition to quitline treatment. Future studies should assess co-use effects on tobacco cessation outcomes and explore combined treatment or bidirectional referrals between quitlines and marijuana treatment providers.

**Summary**

**What is already known?**

Co-use of marijuana and tobacco is common and can negatively affect attempts to quit either substance.

**What is added by this report?**

Our study is the first to present surveillance data from publicly funded tobacco quitlines on the number of callers currently using marijuana.

**What are the implications for public health practice?**

Findings showed that 1 in 4 (24%) quitline callers used marijuana in the past 30 days, and among these, 43% were interested in quitting or cutting down on their marijuana use. Quitlines provide an important source for identifying co-users and finding ways to serve the needs of people who want help to quit these substances.

## Introduction

Smoking tobacco is the leading preventable cause of death in the United States. Although smoking rates have dramatically decreased in recent years in the general population, they have remained high in people with mental health conditions and substance use disorders (1). Marijuana is legal in several states and the District of Columbia, and rates of marijuana use are rising among adults throughout the United States (2,3). From 18% to 39% of adults who used tobacco in the past month in the United States also used marijuana, and 69% to 78% of marijuana users used tobacco in the past month (4,5). Recent longitudinal research showed that marijuana users were more likely to start smoking cigarettes, continue to smoke cigarettes over time (as opposed to quitting), and relapse back to cigarette smoking once they had quit (6). Daily use of marijuana is associated with a 3.6-fold increase in nicotine dependence (7,8).

The most widely used, publicly available, and cost-effective tobacco cessation programs in the United States are tobacco quitlines. They operate in all 50 states and offer a range of services (telephone counseling, mailed materials, cessation medications, and web and text-based interventions) (9–11). Quitlines provide effective cessation interventions, and research has shown a strong dose response with a higher number of calls completed associated with higher quit rates. Combining counseling with medications achieves better tobacco quit rates than either treatment alone (9,12). Because marijuana use may negatively affect tobacco cessation efforts, knowledge of marijuana use among quitline callers and its effect on adherence to tobacco cessation treatment is needed to understand how existing quitline interventions might be adapted to improve outcomes for co-users of tobacco and marijuana. The purpose of our observational study was to describe marijuana use among quitline callers in states with legalized marijuana, including prevalence of marijuana use, characteristics of users, effect of marijuana use on what services quitlines offer callers, and co-users’ interest in reducing or quitting marijuana.

## Methods

**Setting**. Publicly funded quitlines in 2 states (Oregon and Alaska) and the District of Columbia participated in our study. Quitline services in Alaska and the District of Columbia consisted of a multiple-call counseling program and a free supply of nicotine replacement therapy (NRT) products. Uninsured or Medicaid insured callers in Oregon also received multiple-call counseling and NRT. Insured callers in Oregon received a single counseling call and NRT. Quitline counselors in all 3 locations were available 24 hours a day. At the time of data collection (September through December 2016), adult recreational marijuana use (nonmedical use) was legal in all 3 places. Oregon had both a nonmedical and medical marijuana retail marketplace during the entire study period. Alaska’s retail marijuana marketplace opened at the end of October 2016; there was no medical marketplace in the state prior to this. The District of Columbia had only a medical marijuana retail marketplace during the study period. The Western Institutional Review Board reviewed and approved this study.

**Participants**. Callers to the 3 participating quitlines (N = 1,059) who were aged 21 or older, smoked at least 5 tobacco cigarettes per day, spoke English, requested a cessation intervention, reported they were ready to quit tobacco in the next 30 days, and who were not pregnant/nursing were asked the first marijuana use question, “Have you used marijuana in the past 12 months?” (Table 1). Thirty-two of those refused to answer.

**Table 1:** Marijuana Use Among State Tobacco Quitline Callers (N = 1,059) in Alaska, Oregon, and the District of Columbia, September–December, 2016

Study Marijuana Assessment Questions	n (%)
**Q1: Have you used marijuana in the past 12 months?**	
Yes	339 (32.0)
No	685 (64.7)
Refused to answer	32 (3.0)
Don’t know	3 (0.3)
Total	1,059
**Q2: During the past 30 days, on how many days did you use marijuana?**	
0	87 (25.7)
1–19	152 (45.0)
20–29	22 (6.5)
30	70 (20.7)
Don’t know	7 (2.1)
Total	338^b^
**Q3: The next question asks about how you use marijuana. Please tell me all that apply.^a^ Did you:**	
Smoke it (eg, in a joint, bong, pipe, or blunt)	223 (91.4)
Eat it (eg, brownies, cookies, candies)	31 (12.7)
Vaporize it (eg, vape pen or e-joint)	22 (9.0)
Dab it (eg, using a dab rig)	12 (4.9)
Drink it (for example in tea, cola)	7 (2.9)
Other	1 (0.4)
Total	244
**Q4: Would you like to quit or cut down on your use of marijuana?**	
Yes	104 (42.6)
No	128 (52.5)
Don’t know	10 (4.1)
Refused to answer	2 (0.8)
Total	244

a Callers could select more than one, so percentages total more than 100%.

b Does not match “yes” answer to question 1 because of missing data.

**Measures**. We collected self-reported demographic and tobacco use variables from callers at registration with the quitline and used internal quitline data on callers’ treatment adherence (number of counseling calls completed and shipments of NRT). Demographic data collected were age, sex, race/ethnicity, education, insurance coverage, and whether the caller had 1 or more of 4 chronic diseases (asthma, chronic obstructive pulmonary disease, coronary artery disease, diabetes). Tobacco variables were tobacco type, number of tobacco cigarettes smoked per day, time to first cigarette (how soon after waking the caller smoked a cigarette), and use of electronic cigarettes. The 3 participating quitlines asked that we limit the number of study questions we added to standard registration to reduce delay in connecting a caller with counseling. Our 4 study questions, which were adapted from the National Survey on Drug Use and Health (13) and the Behavioral Risk Factor Surveillance System (14), assessed use of marijuana in the last 12 months, frequency of its use in the past 30 days, mode of use (eg, smoking, vaping, eating, drinking), and interest in quitting or reducing marijuana use (Table 1). We defined current marijuana users as callers who reported marijuana use in the past 30 days. We defined nonusers as callers who had not used marijuana in the past 30 days. We defined daily or near-daily marijuana users as those who used marijuana on at least 20 of the past 30 days.

**Analyses**. We used descriptive statistics (frequency, mean, and standard deviation [SD]) to report the individual variables and quitline use data. We used χ^2^ and regression analyses to compare current users with nonusers on demographics, tobacco use variables, and treatment use (counseling calls completed and shipments of NRT), controlling for the state or district. Among current marijuana users, we also compared callers who wanted to quit or cut down on marijuana use with those who did not on demographics, tobacco use, marijuana use, and treatment use. We controlled for state and district because of differences in marijuana policies, quitline promotional activities, and quitline services provided.

## Results

Among the 1,059 study participants, 339 (32.0%) reported using marijuana in the past 12 months and 35 (3.3%) refused to answer or responded “don’t know” (Table 1). We asked callers who reported using marijuana in the past 12 months about frequency of marijuana use in the past 30 days. Of the 339 who answered yes, only 7 responded “don’t know.” Of those who said they used marijuana in the past 12 months, 87 (25.7%) reported no use in the last 30 days. Among the 244 (24.0%) current marijuana users, the mean number of days used in the past 30 days was 14.2 (SD, 12.1); among current users, 37.7% (n = 92) were daily/near-daily users (used marijuana 20–30 of the past 30 days). The primary mode of marijuana use was smoking (91.4%), followed by eating (12.7%) and vaping (9%). Less than half of callers who used marijuana (42.6%; n = 104 users) reported a desire to quit or reduce their marijuana use.

Current marijuana use was lower in the District of Columbia (16.7%) than in Alaska (28.9%) or Oregon (25.0%), *P* = .09. Current users, compared with non-users (n = 772), were less likely to be women (48.4% vs 62.0%, respectively, *P* < .001). (Table 2). We found no other significant differences in caller characteristics based on marijuana use in the last 30 days. Most quitline callers surveyed (96.3%) smoked cigarettes, and only 5.8% used more than 1 tobacco type (eg, cigarettes, cigars, pipe tobacco, or smokeless tobacco). Only 10.0% of callers reported using electronic cigarettes, and this percentage did not differ by marijuana use. Reports of wanting to cut down or quit marijuana were more common among callers from Alaska (54.6%) and the District of Columbia (56.8%) than among Oregon callers (37.9%) (*P* = .03) and more common among callers in the multiple-call program (51.6%) than among those in the 1-call program (40.1%, *P* = 0.03). Callers who reported using edible marijuana (n = 29) were less likely to say they wanted to quit (37.9%) than those who did not use edibles (45.8%) (*P* < .001). Similarly, those who vaped marijuana (n = 17) were less likely to say they wanted to quit (29.4%) than those who did not vape marijuana (46.0%) (*P* = .008). Number of calls completed was not significantly different between those who wanted to quit/cut down on marijuana use and those who did not.

**Table 2:** Characteristics of Quitline Callers in Oregon, Alaska, and the District of Columbia, by Reported Use of Marijuana in the Past 30 Days, September–December, 2016^a^

Baseline Data	Total (N = 1,016)	Current Marijuana Users (used in past 30 days) (n = 244, 24.0%)	Nonusers (did not use in past 30 days) (n = 772, 76.0%)	*P *Value^b^
Age, y				
**Mean (SD) **	**48.6 (14.4)**	**46.5 (14.1)**	**49.3 (14.4)**	**.30**
18–24	53 (5.2)	13 (5.3)	40 (5.2)	.24
25–40	268 (26.4)	79 (32.4)	189 (24.5)	
31–59	478 (47.0)	109 (44.7)	369 (47.8)	
≥60	217 (21.4)	43 (17.6)	174 (22.5)	
**% Women**	596 (58.7)	118 (48.4)	478 (62.0)	<.001
**Race/ethnicity**				
Non-Hispanic White	612 (61.6)	162 (67.2)	450 (59.8)	.45
Non-Hispanic Black	232 (23.3)	40 (16.6)	192 (25.5)	
Non-Hispanic other	150 (15.1)	39 (16.2)	111 (14.7)	
**Education**				
<High school diploma	167 (17.2)	45 (18.8)	122 (16.6)	.46
High school diploma or GED	345 (35.5)	82 (34.3)	263 (35.8)	
>High school diploma	461 (47.4)	112 (46.9)	349 (47.5)	
**Insurance type**				
Uninsured	133 (13.4)	28 (11.6)	105 (13.9)	.52
Medicaid	506 (50.8)	133 (55.2)	373 (49.4)	
Insured + Medicare	357 (35.8)	80 (33.2)	277 (36.7)	
**Has ≥1 of 4 chronic conditions^c^**	414 (41.1)	104 (43.0)	310 (40.5)	.66
**Tobacco Use**				
Cigarette	978 (96.3)	234 (95.9)	744 (96.4)	.81
Polytobacco (uses ≥2 types^d^)	59 (5.8)	18 (7.4)	41 (5.3)	.09
E-cigarettes	101 (10.0)	25 (10.2)	76 (9.9)	.77
Cigarettes per day, mean (SD)	15.3 (10.0)	15.7 (10.1)	15.2 (9.9)	.61
<5 Minutes to first daily use	417 (42.6)	101 (42.4)	316 (42.6)	.96
**Received NRT sent from quitline**	739 (72.7)	167 (68.4)	572 (74.1)	<.001
**Quitline behavior**				
Enrolled in multiple call program	450 (44.3)	100 (41.0)	350 (45.3)	.51
Completed multiple-call program, mean (SD)	2.3 (1.5)	2.4 (1.3)	2.3 (1.6)	.60
Completed 0–2 calls^e^	290 (64.4)	57 (57.0)	233 (66.6)	.005
Completed ≥3 calls	160 (35.6)	43 (43.0)	117 (33.4)	

Abbreviations: GED, general equivalency diploma; NRT, nicotine replacement therapy; SD, standard deviation.

a Values are number (percentage) unless otherwise indicated. Section totals and percentages may vary because of missing data.

b Rao-Scott *P* values from the surveyfreq procedure (SAS Institute, Inc), including state as a cluster variable to account for variability associated with state. Rao-Scott modified χ^2^ *P* values are reported for race/ethnicity and enrollment in the multiple-call program. Comparisons were between current marijuana users and nonusers only.

c Asthma, chronic obstructive pulmonary disease, coronary artery disease, diabetes.

d Cigarettes, cigars or cigarillos, pipes, smokeless tobacco, or other tobacco products. E-cigarettes were not included in the calculation.

e After collecting registration data, the caller is transferred to a coach for their counseling session. Two individuals did not receive any counseling because they chose to be called back at another time and could not be reached.

Current marijuana users were less likely to receive free NRT from the quitline (68.4%) than callers who did not use marijuana in the last 30 days (74.1%) (*P* < .001). Among the 450 participants in the multiple-call program, the mean number of calls completed was 2.4 (SD, 1.3) for current marijuana users and 2.3 (SD, 1.6) for nonusers (*P* = .60). However, marijuana users were more likely to complete 3 or more calls to the quitline than nonusers (43.0% vs 33.4%, *P* = .005) (Table 2).

## Discussion

Tobacco quitlines provide an opportunity to reach tobacco and marijuana co-users, assess their interest in changing marijuana use, and offer tailored services to improve outcomes. Our study provides new information on marijuana and tobacco use among callers to quitlines and confirms that co-use is common (about 25% in states with legalized marijuana) and that many co-users are interested in quitting or cutting down their marijuana use. Quitline callers who reported current use had used marijuana an average of 14 days in the past month. Co-use of tobacco and marijuana has been shown to increase the addiction potential of marijuana (15,16). Furthermore, co-use of tobacco and marijuana complicates efforts to quit either substance. A recent study showed that among 282 co-users, high rates of substitution were reported by those who tried to quit; 50% of co-users increased their marijuana use during tobacco cessation attempts and 62% increased their tobacco use when trying to quit marijuana (17). More research is needed regarding the effects of marijuana use on tobacco cessation outcomes among quitline callers.

Our study found that use of marijuana affected engagement with quitline services in unexpected ways. For example, quitline callers who reported using marijuana were less likely to receive NRT. We saw no obvious reasons why marijuana users would be less likely to receive NRT, which was offered to quitline callers in all 3 of the states/districts participating in the study. Although we saw no difference between average number of coaching calls taken by those who used marijuana and those who did not, those who reported marijuana use were more likely to take 3 or more coaching calls. Only about one-third of callers in our study took 3 or more calls. Only 1 of the states/districts in the study, Oregon, offered text messaging at the time of our study, so those data were not included in our analysis.

A substantial number of co-users calling the quitline to quit tobacco also stated that they wanted to quit or reduce marijuana use (43%). The absence of population-based approaches to marijuana use treatment in the United States presents a potential public health opportunity. Future studies should assess the effect of co-use on smoking cessation outcomes and explore the potential for treatment tailored to co-use to assist with tobacco and marijuana cessation. Tailored treatment of marijuana and tobacco co-users in a quitline setting would need to take the complexities of marijuana use into account, including assessment (eg, medicinal vs nonmedicinal use, modes of use), changing public perceptions of marijuana, varying state laws, and a more limited evidence base on effective marijuana cessation programs. Furthermore, harm reduction models are common in marijuana treatment (rather than the abstinence-based model used for tobacco dependence treatment) and are codified in some state regulations (eg, Washington State [18]).

Our study had limitations. We collected data in only 2 states and the District of Columbia and excluded any quitline caller under the age of 21. All participants were cigarette smokers and stated a willingness to quit tobacco within 30 days. However, characteristics of our study population were similar to the general quitline population (except that the average cigarettes per day and percentage of women were lower than the general quitline population). Other characteristics were similar regarding mean age, percentage of racial/ethnic minorities, and percentage of uninsured participants or those insured by Medicaid (19). Findings may not be generalizable to states that have not legalized nonmedical and/or medical use of marijuana. Given the limited number of questions we were able to add to the standard registration data collection, we could not determine whether participants were using marijuana for nonmedical or medical reasons or both. However, research indicates that many marijuana users, and co-users in particular, may use it for both (20). Oregon had a lower percentage of people wanting to quit or cut down marijuana use. This may have been due to its longer standing and established medical marijuana market. People who use marijuana for medical reasons only may be less likely to want to quit or cut down use. However, nationally representative data suggest that only around 10% of adults report using marijuana for medical reasons only (20). We also do not know whether the co-users we surveyed wanted to quit marijuana or just cut down or if they were using marijuana simultaneously or sequentially with tobacco. At the time of our data collection in 2016, most participants who reported using marijuana (91%) reported smoking marijuana, followed by eating it (13%) and vaping it (9%). Because tobacco use is changing as vaping rates increase, more research is needed on current modes of use, substances used simultaneously or sequentially, and whether mode of use affects cessation efforts. Not all states in this study had open marketplaces at the time of the study, although nonmedical legislation policies were in place. The District of Columbia did not have an open nonmedical marketplace (had only a medical marketplace) at the time of our study, which could have contributed to our finding lower rates of use there than in Oregon and Alaska. Future studies should address these limitations.
